# Advancing Drug Discovery Through Mechanistic Validation and Translational Evaluation

**DOI:** 10.3390/biomedicines14102401

**Published:** 2026-10-21

**Authors:** Manal A. Nael, Laxman M. Alakonda, Khaled M. Elokely

**Affiliations:** Division of Pharmaceutical Sciences, School of Pharmacy, College of Health Sciences, University of Wyoming, Laramie, WY 82071, USA

The Special Issue “Advances in Novel Drug Discovery, Synthesis, and Evaluation” brings together five original research articles and one narrative review examining how experimental interventions might become useful therapies. The contributions span small molecules, supramolecular formulations, antigen-encoding messenger RNA, and shared mechanisms of chronic pain. Across these approaches, a recurring challenge is to connect biological activity with a causal mechanism and a meaningful disease outcome. The collection advances this task through complementary experimental strategies, while identifying questions that remain before clinical translation. Its central contribution is an opportunity to examine how evidence from synthesis, formulation, and biological evaluation can inform the next stage of therapeutic development.

This challenge extends across drug discovery. Exposure at the site of action, target binding, and functional pharmacological activity provide an established framework for interpreting whether a candidate has been adequately tested [1]. Delivery technologies add another dimension because the location and context of exposure can determine the biological response. For example, noninflammatory mRNA formulations have supported antigen-specific immune regulation in experimental autoimmune disease [2], and changes in lipid nanoparticle composition have enabled selective organ targeting [3]. These developments encourage closer integration of chemistry and delivery science with disease biology. They also make it necessary to distinguish successful delivery or pathway modulation from evidence that an intervention changes disease.

Two contributions examine how pharmacological intervention affects fibrosis in different settings. Yang et al. investigated the MyD88 inhibitor TJ-M2010-5 in bleomycin-induced pulmonary fibrosis and cultured human lung fibroblasts [4]. Reduced fibrotic endpoints were accompanied by changes in autophagy-associated measurements, and pharmacological interference with autophagy attenuated the protective effects. The inclusion of delayed treatment, alongside preventive administration, strengthens the rationale for further evaluation. In a murine model of triple-negative breast cancer, Ramírez-Farías et al. examined the valproic acid derivative N-(2-hydroxyphenyl)-2-propylpentanamide [5]. Changes in collagen deposition, fibroblast-associated markers, and GPER1 expression suggest opportunities to investigate the tumor stroma as a pharmacological target. These stromal changes were not accompanied by a significant reduction in tumor growth. Together, these studies motivate further work on tissue remodeling, including whether modifying the microenvironment can improve responses to established treatments.

Formulation development offers a complementary route to improving a therapeutic candidate. Khantakova et al. prepared a vortioxetine–glycyrrhizic acid complex by mechanochemical processing and characterized its physicochemical and biological properties [6]. Improved solubilization and transport across a Caco-2 monolayer were accompanied by changes in microglial responses and peripheral blood-cell measurements. These findings connect material preparation with experimentally measurable pharmacology. Because the animal experiments involved short treatment in intact mice, the results provide a basis for investigating pharmacokinetics and antidepressant activity in disease models. They also raise an experimentally tractable question about the respective contributions of the carrier and the assembled complex.

Arai et al. explored a different relationship between delivery and biological response by administering ovalbumin-encoding mRNA in lipid nanoparticles [7]. The study reported changes in Th2 cytokine production and regulatory T-cell populations in mouse experiments, supporting further investigation of antigen delivery for immune modulation. However, the mRNA-LNP intervention did not improve lung histopathology in the airway inflammation model. The separation between immune readouts and tissue pathology is informative for subsequent development. It directs attention to the magnitude and durability of tolerance, the cells receiving the antigen, and the relationship between delivery-associated inflammation and antigen-specific effects.

Whole-organism evaluation can reveal responses that are difficult to anticipate from a single molecular target. Rajpurohit et al. combined developmental observations, histological assessment, an inflammatory reporter, and single-cell transcriptomics to investigate the quaternary ammonium silane K21 in zebrafish [8]. Reported growth and developmental changes, together with reduced LPS-associated NF-κB reporter activity, generate hypotheses about its biological effects. This breadth of observation illustrates the value of integrating phenotypic and molecular measurements during early evaluation. Establishing causal mechanisms and relevance to human disease will require controlled follow-up studies that account for exposure and the aquatic environment.

The review by Duong and Ramdass considers ATP–P2X3 signaling as a possible shared contributor to neural sensitization in endometriosis and irritable bowel syndrome [9]. Its discussion of neuroimmune interactions and cross-organ sensitization provides a rationale for investigating common therapeutic targets across overlapping pain conditions. Clinical experience also defines the limits of that rationale. In the phase 2b SCHUMANN trial, eliapixant did not demonstrate a significant benefit over placebo for the primary endometriosis-associated pain outcome, and the study was terminated prematurely because of liver safety concerns [10]. Early termination constrains efficacy interpretation, and the result should not be generalized to every P2X3 antagonist. It nevertheless emphasizes the need to establish benefit and tolerability in the intended indication, even when the biological rationale is persuasive.

The next phase of research should begin with more precise tests of mechanism. Changes in transcript or protein abundance can identify responsive pathways, but their functional contribution requires additional evidence. Selective chemical probes, suitable inactive controls, and genetic perturbations can help distinguish target-dependent effects from other pharmacological actions [11]. In studies linking autophagy to protection, multiple complementary assays should assess pathway dynamics and marker abundance, following established guidance on autophagy measurement [12]. These approaches would help determine which molecular changes are necessary for benefit and which accompany a broader response. Such distinctions are particularly important when a compound affects interconnected pathways or several cell populations.

Recent structural work provides an example of how mechanistic evidence can guide chemical refinement. Zhao et al. characterized the human P2X3 receptor in relation to sivopixant binding and subtype-selective inhibition [13]. Structural information of this kind can support the design of compounds with defined receptor interactions and inform subsequent pharmacological testing. For candidates arising from the Special Issue, the corresponding priorities include measuring target engagement in relevant cells or tissues, determining activity across related targets, and connecting those measurements with exposure. Computational predictions can help prioritize experiments, but advancement should depend on experimentally demonstrated performance. The translational value of greater selectivity depends on its consequences for efficacy and safety.

Exposure–response analysis should therefore become an early component of development. Greater aqueous solubilization or transport through an epithelial model does not by itself establish improved systemic availability or delivery to the diseased tissue. Formulation studies should connect dissolution, stability, and permeability with measured concentrations of the active compound and, where relevant, its metabolites. For central nervous system applications, brain exposure is especially important. Dose selection should then be related to pharmacodynamic effects and tolerability. This would allow differences between formulations to be interpreted in terms of exposure, intrinsic activity, or both, and help identify conditions under which a promising laboratory effect remains achievable in vivo [1].

The formulation itself also requires careful experimental separation. When a carrier has biological activity, comparisons should include the carrier alone and an uncomplexed mixture, alongside the assembled formulation and the parent drug. This design can distinguish a delivery advantage from additive or synergistic pharmacology. For nucleic-acid systems, biodistribution should be resolved at the cellular level and linked to the amount and duration of antigen expression. A further priority is to determine whether the formulation induces inflammatory responses that modify the intended immune effect. The experience with noninflammatory mRNA and organ-targeted nanoparticles provides useful precedents; each new formulation still requires direct characterization [2,3].

Progress will also depend on choosing outcomes that test the proposed therapeutic purpose. Cytokine modulation should be connected to sustained improvement in tissue pathology or organ function. Altered tumor fibrosis should be evaluated alongside tumor control, drug penetration, and response to combination treatment. In neuropsychiatric applications, molecular and immune findings should be integrated with appropriate behavioral outcomes and pharmacokinetic measurements. When repurposing a target across pain conditions, trials should address disease-specific symptoms and functional impairment. Prospective studies can test whether pathway-associated biomarkers identify responsive subgroups, while avoiding the assumption that a mechanistic signature necessarily predicts clinical benefit. Negative and discordant results should guide refinement of the hypothesis and endpoint selection.

Complementary human models can improve the relevance of this evaluation. Primary cells, patient-derived systems, and microphysiological models should be selected for the particular disease process or safety question being investigated. The performance assessment of a human Liver-Chip by Ewart et al. illustrates how a model can be tested against a defined set of compounds with known clinical toxicity profiles [14]. Such evidence supports evaluation for a specified use, rather than assuming that greater model complexity guarantees predictive value. Replication across appropriate systems, including models that capture chronic disease, can help reveal whether an effect depends on an experimental setting that will not be reproduced in patients.

Safety assessment should develop alongside efficacy testing. Short observation periods or a limited set of organ measurements cannot characterize the consequences of repeated exposure. The duration and scope of evaluation should reflect the intended clinical use, particularly for chronic conditions. Immune-modulating interventions require attention to host defense functions, while formulations require assessment of carrier-related effects and those of the active agent. Reproducible development also depends on transparent experimental design. Prespecified outcomes, justified sample sizes, randomization, blinding, and clear reporting of exclusions and attrition allow findings to be assessed and repeated. The ARRIVE 2.0 guidelines provide a practical basis for improving these aspects of animal research [15]. Accessible protocols and appropriately shared data further support independent evaluation.

The contributions to this Special Issue identify several opportunities for continued investigation and show how different experimental approaches can inform one another. Their greatest value for future discovery lies in the questions they make possible to test. Integrating chemical preparation and delivery with causal biology, exposure measurements, and meaningful disease outcomes will help determine which interventions merit further development. Sustained attention to safety and reproducibility will make those decisions more informative. I thank the contributing authors and reviewers for the work that made this collection possible and for advancing the discussion of how experimental findings can be developed into credible therapeutic opportunities.

## Data Availability

No new datasets were generated or analyzed in preparing this editorial. The discussion is based on published literature cited in the manuscript. Data sharing is not applicable to this article.
